# “Irresponsible Not to Share Such Important Information”: A Narrative of Responsibility That Underpins Familial Disclosure of Genetic Risk in Singapore

**DOI:** 10.1111/bioe.70067

**Published:** 2025-12-07

**Authors:** Serene Ong

**Affiliations:** ^1^ Centre for Biomedical Ethics, Yong Loo Lin School of Medicine, National University of Singapore Singapore

**Keywords:** familial disclosure, familial hypercholesterolemia, genetic risk, responsibility

## Abstract

Patients diagnosed with familial hypercholesterolaemia (FH) are advised to disclose their diagnosis to at‐risk relatives, as early knowledge of genetic risk allows relatives to manage their cholesterol levels. Debates in the bioethical literature regarding familial disclosure of genetic risk tend to revolve around rights: the right of the patient to autonomy and confidentiality, against the right of the family members to receive information that is clinically relevant or genetically shared. However, empirical studies found that patients' reasoning was frequently relational, suggesting a need to reexamine the values used in guidelines. This study aimed to examine patient values that affect the familial disclosure of familial hypercholesterolaemia. Twenty‐one patients were interviewed. The results showed that patients' decisions were driven by two factors: beneficence and emotional proximity. As interview questions did not ask specifically about responsibility, it is noteworthy that various notions of obligations to others emerged unprompted. Patients perceived responsibilities based on the knowledge they held, and the nature of familial relationships; in particular, duties to vulnerable relatives. My results suggest that responsibilities, rather than rights, are a decisive moral issue that motivated patients' decisions.

## 1 Introduction

When a patient tests positive for familial hypercholesterolaemia (FH), clinicians often recommend informing at‐risk family members. As cholesterol build‐up starts from birth, early awareness of genetic risk allows FH carriers to seek genetic testing and treatment. This is crucial as FH significantly increases the risk of a fatal heart attack by age 40—up to 100 times that of the general population [1]. Fortunately, FH is highly treatable with statins, which have good safety profiles [2], and lifestyle modifications such as exercise and diet. Sufficiently early treatment with statins can lower cardiovascular risk to levels comparable to the general population [3].

The clinical factors thus lean towards familial disclosure, and generally, patients report that they would disclose genetic risk information to their relatives [4]. A discordance, however, lies between the normative assumptions embedded within current regulations and empirical evidence. Current approaches to familial disclosure are largely individual‐centric, justified on the patient's rights to privacy and personal autonomy, and regard genetic data as personal and protected data [5, 6, 7, 8]. While most guidelines acknowledge that familial interests are entangled with the results from a patient's genetic test—in rare cases, may even outweigh the patient's interests—they generally mandate that healthcare providers only disclose patient information to at‐risk family members with the consent of the patient [9]. In this framework, the interests of relatives are noted in the form of genetic risks, but only the patient holds explicit rights over the information. Family members, even if directly affected by the information, do not have corresponding rights to access it. Indeed, if patients have not provided consent to share their information before they die, the privacy and autonomy of dead patients are often upheld over potential benefit to living relatives [10].

Yet empirical studies have shown that patients' reasoning for disclosure is frequently relational in nature [4, 11, 12, 13], challenging the individual‐centric paradigm that underpins most current disclosure frameworks. Patients frequently consider the implications for relatives [14] and are influenced by emotional proximity rather than genetic relatedness [4]. In addition, environmental factors, such as access to specialists or health insurance coverage for genetic testing, can either facilitate or hinder familial disclosure [15]. Cultural context also plays a significant role, with ethnic norms and values shaping whether and how individuals communicate about health conditions within families [16]. In other words, patients often approach disclosure through a *relational lens*, weighing the well‐being of their family members, the quality of their relationships, and the emotional and social dynamics at play. For example, patients are more likely to disclose when there is clear medical benefit for the recipient [17, 18], but may withhold information if they anticipate emotional harm [19], believe the relative lacks the capacity to understand [20], or assess the risk of transmission as low based on the relative's life circumstances (e.g., being childless or unmarried) [21]. Disclosure also occurs along lines of affection [4, 22] and is complicated by family dynamics [19]. Finally, disclosure is shaped by situational factors external to the patient's choices. For example, the patient may also be concerned that the information will disrupt the relative's marriage plans [22].

This suggests a misalignment between the individual‐centric justifications underlying clinical guidelines for familial disclosure and the reasoning used by patients who have faced this dilemma. For example, Singapore guidelines for clinicians on disclosure of genetic information to family members generally recommend that the patient's decisions be respected, but there are provisions for disclosure without consent [23, 24]. These policies treat genetic information as the personal property of the patient, placing a strong emphasis on confidentiality and individual rights. This discrepancy might be problematic for patients and clinicians as guidelines that feel disconnected from patients' lived experiences, especially in cultural contexts where familial obligations are deeply embedded. Identifying the actual values and considerations that influence patients' disclosure decisions might inform revisions of these professional guidelines to be more consistent with the lived experiences of patients. As Wilson had argued, he wrote that arguing based on rights results in an impasse, and alternative approaches based on other values, such as benefits and harms of genetic knowledge, may offer more productive paths forward [8].

Focusing on guidelines is especially important because, although policy advisory bodies that issue such guidance are not executive entities and do not hold direct regulatory or supervisory authority, their recommendations nonetheless shape clinical practice and ethical norms. In Singapore, for instance, the Bioethics Advisory Committee (BAC) Reports have been accepted by the Ministry of Health (MOH) as providing authoritative guidance on ethical matters not explicitly governed by statute or subsidiary legislation [25]. This gives guidelines significant practical influence, particularly in areas such as familial disclosure where legal mandates are sparse.

It should also be noted that the current approaches outlined above represent a generalised view of most international guidelines, and there are important local variations and nuances across countries, jurisdictions, and over time. For example, before 2011, France had some of the strictest confidentiality regulations, prohibiting healthcare providers from directly contacting at‐risk family members unless they had specific professional justifications [26]. However, in 2011, legislation was introduced that imposes a legal obligation on patients to inform at‐risk family members and allows patients to authorise healthcare providers to disclose genetic information to relatives if they prefer not to do so themselves [27].

Despite a wide body of research on patients' reasoning for disclosure, empirical data on patients' disclosure reasoning remain scarce in many parts of the world, particularly in Asia. This is significant because local sociocultural norms, familial structures, and sociopolitical and legal contexts may shape distinctly different attitudes towards genetic information, influence relationship dynamics around familial disclosure, and affect social and ethical considerations regarding privacy and consent. Addressing this gap is thus crucial for developing contextually relevant guidelines and policies.

This study examines the values and considerations that shape how patients in Singapore decide whether and how to disclose FH risk to their relatives. FH, which has been identified as a Tier 1 genetic disease by the U.S. Centres for Disease Control [28], as FH is common enough to be a burden, and early detection and intervention could notably reduce morbidity and mortality. While epidemiological data on FH are lacking in the Asia Pacific, FH is estimated to be prevalent and underdiagnosed [29].

## 2 Methods

The findings reported in this study were part of a larger research project, ‘Disclosure of Lynch Syndrome and Familial Hypercholesterolaemia Genetic Risk Information to Family Members’, which was conducted in accordance with approvals obtained from the Singapore NHG Domain Specific Review Board (DSRB) (2020/01290).

I recruited FH patients over 21 years old between March 2021 and July 2023 at the specialist FH clinic at the National University Hospital Singapore. During their clinical visit, patients were informed of this study by their attending clinician and invited to participate. I was stationed in a room adjacent to the examination room. If patients expressed interest, I would explain more about the project and give them a study information sheet. Informed consent was taken at the time of recruitment. An interview was then scheduled for a later date. As interviews were conducted some time after recruitment, a few patients changed their minds and chose to withdraw. These patients were followed up by phone twice, 2 to 3 weeks after initial contact, before being regarded as having withdrawn from the study.

Semi‐structured interviews were conducted to obtain rich descriptions and identify previously unreported issues (Appendix A). The interview questions explored patients' experiences of familial disclosure. The interview guide was reviewed by two colleagues not involved in the project and adjusted for flow and understandability based on their feedback. Interviews were conducted individually in English, with participants given the option to be interviewed in person, over the phone, or via Zoom. All interviews were audio‐recorded and transcribed verbatim. I used inductive content analysis [30] to search for themes relevant to the research question. The analysis was inductive, with codes identified during the coding process rather than *a priori*. Similar codes were then grouped into broader categories to identify patterns. From these categories, I abstracted overarching themes that captured the core values underpinning how and why patients made disclosure decisions. Coding was done iteratively as interviews were conducted to allow for adjustments to the guide as interviews progressed, and to identify saturation. Saturation was defined as two consecutive interviews with no new themes identified [31]. Note that precise quantification of issues raised is not possible. Instead, the themes raised from data analysis were meant to identify and inform pertinent topics around familial disclosure of genetic risk.

## 3 Results

Twenty‐three patients were recruited, and 21 were interviewed (response rate 91.3%). Two participants were lost to follow‐up after recruitment. Patient demographics are shown in Table 1. Three themes were identified. The first two themes were key factors that shaped patients' decisions: beneficence to family members and emotional proximity. While patients reported a variety of reasons for disclosure, many of these reasons can be collapsed into these two main motivations. The third theme was the notions of perceived responsibilities that emerged, either arising from the knowledge the patient held (a general moral responsibility) or the emotional relationship with a relative (a special responsibility). All patient names used in this article are pseudonyms.

**Table 1:** FH patient demographics.

	Patients (*N* = 21)
Median age, range (yrs)	44, 21–63
Gender	
Female	14
Male	7
Ethnicity	
Chinese	19
Malay	0
Indian	2
Have children	
Yes	14
No	7

### 3.1 Beneficence

Beneficence, the principle to act for the benefit of others, featured prominently in interviews. Both patients and family members recognised that genetic risk information would enable family members to be informed about their own health risks and take preventive measures. They felt that early detection of FH and treatment was important; for example, Irene (F, 28 years) called it ‘a ticking time bomb’. Patients often framed disclosure in terms of health‐related benefits, explaining that it would be better to ‘get the treatment earlier rather than later’ (Diana, F, 32 years).

I'd rather know something than have a knowledge gap … So that you can identify something earlier and tackle it rather than … [when] it's too late.

Qiang Zhang, M, 32 years

Of note was that patients took a wide interpretation of benefit. While the clinical value of the information was often central to their reasoning, patients also pointed to psychosocial, educational, and behavioural dimensions. Some patients saw merit in the learning value of the information and cautionary advice, even before a formal diagnosis. For instance, Oliver (M, 47 years) informed his cousins before his genetic test results were returned, advising them to check their cholesterol and adhere to medication: ‘Don't be like me, [only] when I remember I take [my medicine].’ Some patients said they discussed their experiences with friends because they felt that the discussion was important from ‘an educational point of view’ (Irene). Their stories reflected a broader view of disclosure as a social act, not limited to those at genetic risk.

Notably, patients highlighted the benefits of the increased agency knowledge provided. Recognising that genetic risk information presented a form of foreknowledge, some patients spoke of being able to make different choices in life as a result of the information, including career, lifestyle, or reproductive choices. Qiang Zhang gave an example: say his child tested positive and could not enter a particular career because of the career demands. Rather than view the situation as one of constraint or discrimination, he said, with early knowledge, his child could mentally prepare not to even consider that career, rather than put in years of training only to realise that the career was not suitable for them.

It's always good to know. At least you have the chance or the opportunity to, to do something like to prepare yourself or to finish things that you need to do.

Fiona, F, 59 years

Conversely, if patients did not perceive the information as beneficial for their relatives, they would be less inclined to disclose. A key factor influencing this perception of beneficence was their risk perception of FH.

The information I get about this condition doesn't sound very dangerous … it can lead to cardiac arrest and stuff, but things like blockage and all if you didn't get it treated or have something going on to help maintain the level, but it doesn't sound very life‐threatening.

Diana

The sentiment was not unique to Diana; a number of patients viewed FH as low risk, even after being informed by their clinician that high cholesterol levels can lead to cardiac arrest. Even after a probable FH diagnosis, Claire (F, 56 years) asked, ‘Is it very serious?’ She further reflected that the absence of physical pain contributed to people's perception of the condition's severity.

I think human beings, if it affects them physically, the pain comes, then they would think about how to resolve the pain.

Claire

These comments suggest that patients' decisions about disclosure are not driven solely by medical risk assessments, but also by subjective perceptions of harm and urgency. When FH is not experienced as an immediate or tangible threat—particularly in the absence of symptoms—its salience diminishes. As a result, the moral imperative to share potentially life‐saving information may be overridden by more immediate psychological and relational considerations.

### 3.2 Emotional Proximity

Emotional, or affective, proximity between the patient and family member was the other strong driver for disclosure. Many patients reported telling their partner first—individuals they were most emotionally close to, but who did not carry a genetic risk. As well, many patients specifically highlighted their relationship with family members as a reason for disclosure, suggesting that certain familial bonds carried a special weight distinct from other types of relationships.

You will want to protect your family members … I guess that's when people will be willing to share.

Diana

Patients' notion of ‘closeness’ was primarily defined by affective and not genetic distance. For instance, Irene told one cousin because ‘she's a best friend’, but not with other cousins because ‘we are not on the texting kind of closeness’. Qiang Zhang said he would not disclose to his second‐degree relatives because he does not ‘really talk to them’ adding, ‘let's say like we're quite close, I don't see why not’.

Notably, all of the patients said they had known they had high cholesterol for some time—five patients reported awareness of it for over 20 years—before receiving a formal FH diagnosis. Throughout their ‘medical journey’, many had kept immediate family members updated on their health, and the diagnosis of FH was often regarded as just another update. A number of patients used the phrase, ‘there's nothing to hide’ (Anna, F, 59 years), to indicate the transparency that characterised their relationships with close family members.

For such close relationships, disclosure was less about the content of the message (the genetic risk information) and more about the process of communication. Disclosure to close family members was driven by personal reasons such as trust and affection, while subsequent disclosure to distant relatives was motivated more explicitly in terms of beneficence.

I have some closer cousins whom I will probably share that I have this high cholesterol issue with regardless of the FH diagnosis … but some of the more distant, not so close cousins … I would probably only share with them if there is a positive FH diagnosis.

Grace, F, 30 years

Rather than sharing information solely based on medical relevance, patients also considered relational ease and the likelihood of productive discussions when deciding whom to inform. In some cases, this meant that two relatives with the same level of genetic risk were treated differently: one was informed, while the other was not, due to differences in the quality of the relationship or perceived openness to the information. For instance, Grace said, ‘I have two sisters, but I only told one because I'm a bit closer to her.’ She explained why she did not inform the other sister:

She tells me a lot of the pseudoscience nonsense… And when you want to engage in an argument, it takes your energy right? … like, pick your battles.

Grace

The lack of a close relationship, therefore, presented a barrier to disclosure. Patients interviewed sometimes spoke of whether the information was even appropriate to tell distant relatives. As genetic risk information stemmed from the patient's illness, some patients considered that information personal, and they ‘shouldn't be saying these things to [distant relatives]’ (Irene). Patients felt that for distant relatives, casual topics were acceptable, but they should ‘not really go too in‐depth in [their] personal lives’ (Simon, M, 35 years).

Their comments showed that patients were mindful of the relational dynamics during the disclosure process. They were also concerned about the effectiveness of communication. Hannah (F, 35 years) cautioned that if genetic risk information was communicated to relatives whom they generally did not speak to, the information might ‘cause a bit of panic if [they] didn't convey this thing properly’. They pointed out that the same information, coming from different individuals, will carry a different impact.

Relationship matters in whom you are conveying this message to, even despite it being genetic.

Hannah

The importance of emotional proximity is emphasised when relationships are poor or antagonistic. Estranged from her three sons, Julia (F, 63 years) refused to inform them or provide consent for her clinician to inform them on her behalf. When asked if it is acceptable if someone else informs her sons of their genetic risk, while keeping her identity confidential, she refused as well. It was evident during the interview that her focus on her strained familial relationships influenced not only the way she made her decisions, but the way she interpreted the issue. When I asked about the possible clinical benefit of the information to her sons, she reframed the issue entirely. She insisted that it was not about her health or the family members' right to know; rather, disclosure was a ‘family issue… it's not an open right kind of thing.’ For Julia, disclosure was not a question of beneficence or moral duty; it was grounded in personal history and emotional estrangement.

### 3.3 Implicated Responsibilities

A notable theme that emerged from the interviews was how patients' reasoning was frequently framed in terms of responsibility. This is analytically significant because the interview questions were not explicitly designed to probe for notions of responsibility, yet participants independently mobilised this concept to articulate their motivations. This suggests that responsibility functions as a salient moral lens through which patients interpreted and justified their actions.

In recognising the various ways genetic risk information can impact their family members, patients perceived a sense of responsibility that arises from the knowledge they held—this refers to a general moral responsibility. They described the information as ‘life‐saving’ and felt that withholding it would constitute a moral failure.

If there is a positive diagnosis I feel a little bit responsible that I have this information that can be life changing for [relatives] … irresponsible not to share such important information with them.

Grace

Patients also perceived special responsibilities arising from specific familial, such as parent and child, as sources of heightened responsibilities. Parental duties were especially prominent. Qiang Zhang explained, ‘as a parent I'll be responsible for my kids' well‐being.’ They mentioned wanting to take the genetic test so they could be better informed for their children's sake. Oliver pointed out that his treatment regime would not change regardless of his test result; instead, his decision to undergo testing was framed as a moral duty to protect his children's future.

We bring them into this world… I want to know whether I have passed this bad gene down to them.

Oliver

However, perceived responsibility does not always lead to disclosure. In some cases, patients did not tell their parents because they did not want their elderly parents to worry. Fiona framed nondisclosure as a ‘kind of respect like you shouldn't [disclose]’, unless their older relatives asked specifically about the patient's health. They viewed the responsible action as one that does not worry their parents.

We also don't tell my parents—because we will be the one to know whether parents will be over‐worried.

Paul, M, 62 years

Their accounts highlight a key point: moral responsibility was not always oriented toward maximal transparency or clinical benefit, but toward maintaining emotional equilibrium and respecting perceived relational boundaries.

Patients were often navigating complex tensions between the desire to act beneficently and the realities of their emotional relationships. This was particularly evident in how they handled disclosures to more distant or challenging relatives. While many acknowledged the potential medical benefit of sharing the information, their decisions were equally shaped by relational dynamics and personal comfort. For instance, patients frequently said they felt uncomfortable communicating with the older relatives. In such cases, they often delegated the task of informing extended family members to those with closer ties. Diana said, ‘If I'm not on good terms with [my cousins], then probably I will let my mom inform the family’. This strategy allowed patients to maintain their personal boundaries while still ensuring that information reached those who might benefit—indirectly fulfilling their perceived responsibility.

I think most people will need to unpack their personal feelings and separate it from the benefit to [the relative's] health.

Grace

Indeed, when discussing a hypothetical scenario whereby the patient does not disclose even if the information could benefit relatives, some patients justified a possible breach of confidentiality if it meant safeguarding a family member's health.

If I don't tell [my daughter] … I think that the medical experts … are obliged to tell her because of her medical health.

Qiang Zhang

These views suggest that responsibility is not a static moral category but one shaped by contextual factors: family dynamics, role obligations, and perceptions of vulnerability. The case of Julia, who was estranged from her sons, underscores this complexity. When asked about her reasoning not to inform her sons, she framed the issue as one of reciprocity and familial obligations. She said that after she raised her sons as a single parent following her divorce, they failed to reciprocate with signs of affection or respect, and she felt ‘as good as dead’ to them. In her view, familial obligation is reciprocal. She said, ‘I disown my three sons’, and felt absolved of any moral duty toward them. Julia's stance reveals how responsibility is often conditional upon relational history.

## 4 Discussion

The study explored the values and reasoning that shaped patients' decisions to disclose FH risk to relatives in Singapore. The findings revealed that patients were motivated to disclose for their family members' benefit, their emotional closeness, and a sense of responsibility. In addition, the interview questions aimed to elicit patients' reasoning behind their decisions and actions, but did not ask specifically about responsibility. It is, therefore, noteworthy that various notions of obligations to others emerged *unprompted* from the interviews. This suggests that patients approach genetic disclosure not primarily through a rights‐based lens, as is dominant in the literature [5, 8, 32], but rather through relational and responsibility‐based reasoning.

This study had two limitations: the self‐selection and self‐reporting of informants, and an imbalance of demographic categories. First, as the participation was voluntary, there may be an imbalance towards informants who had positive experiences and/or were inclined to disclose. Individuals who had negative experiences and/or held views contrary to social norms might decline to participate; this was the ‘group not studied’. Second, though recruitment efforts aimed to be broadly inclusive, there were imbalances in gender and ethnicity, two demographics that are related to patient health experiences [33]. Male patients, and Malay patients were underrepresented.

Two key factors were found to motivate disclosure: beneficence and emotional proximity. These factors also generated notions of implied responsibilities. Patients perceived responsibilities arising from the knowledge they held (grounded on the principle of beneficence), as well as special responsibilities arising from familial relationships (shaped by the emotional proximity they hold with the other). Of note was the way these two factors interacted, particularly in cases of conflict: what often guided the resolution of conflict was perceived responsibilities towards the other, which sometimes entailed the need to put aside personal preferences. For example, some decided to disclose despite their discomfort, believing it was in the best interest of the relative. Conversely, others opted not to disclose if they anticipated the information might cause undue anxiety or harm.

While patients mentioned rights, such as privacy rights or the right of family to know, there was a strong sense that obligations were more frequently discussed. Patients' reasoning was frequently focused on others, and whether explicitly stated or implicitly implied, decisions regarding disclosure and nondisclosure were often justified based on perceived family best interests. This framing moves beyond the confines of conventional bioethical discourses that centre on individual autonomy and informed consent. Instead, it underscores a relational ethical perspective, where knowledge and/or relationships are seen to confer responsibility—particularly, when it holds consequences for the lives and futures of others. In this view, responsibility is not a secondary concern to autonomy; rather, it is an intrinsic moral response to the asymmetry of knowledge and risk within families. Even when patients noted that family members have a right to know, their views implied a corresponding obligation on the patient to uphold that right. These findings suggest that responsibilities, rather than rights, are a decisive moral driver that motivated patients' decisions.

These accounts illustrate how genetic knowledge is often situated within moral economies of kinship, where to know is not just to act on one's own behalf, but to act for others. The knowledge becomes imbued with ethical weight because of the relational roles that patients occupy.

At this point, it is worth taking a broader look at the sociocultural background of Singapore to understand how two pre‐existing cultural characteristics have shaped a narrative of responsibility that permeates social and ethical norms. The first characteristic is the role of the family. Social policies in Singapore are explicitly based on the family as the main caregiver. While the primacy of the family is not uniquely Singaporean, these national policies reflect prior social prioritisations of the family [34]. For instance, when discussing social policies on caregiving for the elderly, Mehta [35] wrote that ‘[t]he Singapore public has been constantly reminded that the family should be the older person's first line of support’, followed by the community and then state. Consequently, Singaporeans are used to families as a main pillar of support for individuals. From housing to healthcare, the ‘familialist’ social policies have engineered a population that views the family unit as self‐reliant [36]. This mindset also creates an emphasised expectation of personal responsibility towards family, as family members are expected to take care of each other.

The second characteristic is a sociocultural norm of responsibility. In a commentary on early policy decisions that heavily influenced Singapore's social environment, Haskins [37] noted a consistent emphasis on individual and social responsibility in policies ranging from education, housing, pensions, and healthcare. Sun [38] wrote, ‘[Singaporean] citizenship is primarily interpreted to confer ‘duties’ by the state’. Thus, while social policies reflect an existing social prioritisation of the family, they also reinforce a narrative of responsibility and care towards family, as an extension of responsibility towards self. Policies such as ‘Ageing in Place’ and ‘Many Helping Hands’ to cope with an ageing population place the responsibility of care on the elderly and their families, and indicate explicitly that the family is expected to shoulder the care of their members [39, 40]. More recently, a study of the Hospital at Home (HaH) care model for COVID‐19 patients [41] found that family members willingly took on additional caregiving duties, as compared to HaH studies in other countries. This acceptance was evidenced as well in the ways patients were motivated to disclose: the main driver for disclosure was beneficence, which grounded a forward‐looking sense of responsibility to handle that information.

While concepts such as beneficence and responsibility may be present in other cultures, particularly in collectivist societies in Asia [42], this study contributes empirical evidence and insights into how cultural and policy contexts interact to influence disclosure norms. These findings have practical implications for policy development, particularly in tailoring disclosure guidelines in the familial disclosure of FH risk to align with local sociocultural expectations. FH represents a particularly compelling case for cascade testing initiatives, given its high prevalence, clear heritability, and clinical actionability. The lack of painful or explicit symptoms often leads to delayed diagnosis or underdiagnosis, heightening the ethical significance of proactive disclosure within families. Currently, attending clinicians and genetic counsellors encourage patients to consider familial disclosure, but the rationale and moral weight of responsibility are often left implicit. Making these expectations more explicit, while remaining sensitive to familial dynamics, could help align clinical guidance with local norms. Given the cultural emphasis on familial responsibility in Singapore, patients might be particularly receptive to a duty of beneficence, especially towards their family members. Further work should explore the practical and moral boundaries of familial disclosure responsibilities for the wider public in the specific context of FH: under what circumstances do they feel disclosure is morally required or permissible to withhold? How do they weigh competing obligations? Anchoring such questions in the lived realities of FH will help ensure that ethical guidance remains both contextually relevant and practically usable.

## 5 Conclusion

The extant literature indicates that the discourse around familial disclosure includes important relational and responsibility‐based perspectives; however, regulatory and policy frameworks in practice remain largely grounded in individual‐centric justifications. This study extends and deepens these existing discussions by offering insights into how patients in Singapore navigate the ethical and relational complexities of familial disclosure in the context of a heritable condition like familial hypercholesterolaemia (FH). By centreing patients' lived experiences, the findings highlight that decisions about disclosure are shaped not only by clinical risk assessments but by relational values such as beneficence and emotional proximity. The senses of responsibility that patients feel toward their family members are both culturally embedded and socially reinforced, suggesting that disclosure decisions are often moral responses to familial obligations rather than assertions of individual autonomy. These insights underscore the importance of culturally responsive policy frameworks that make space for local moral reasoning and relational dynamics. As Singapore and other countries expand genetic testing and cascade screening programmes, incorporating patients' values and perceptions into clinical guidelines will be crucial for ethically sound and socially attuned healthcare practices.

## Data Availability

Data that support the findings of this study are available on request from the corresponding author. The data are not publicly available due to privacy or ethical restrictions.

## Appendix A Interview questions

**Introduction**

Can you tell me a bit about yourself?

- Age, family members
  **Topic 1: At the clinic**
  1a. The process
  Can you tell me (roughly) how long ago you were diagnosed with FH?
- Can you walk me through how you were informed?
- What was your reaction? How did you feel?
- Have you taken the genetic test? Why?

What do you think of FH? What does this term mean to you?

When you were told of your genetic test results, you were also told that some of your family members might have the same condition, and that you might want to inform your family members of their risk. Can you describe your experience?

• When were you informed that your genetic test results may affect your family members, directly as they may also carry the same condition’?
• Who were involved and present during the clinical session(s)?
  ◦ Did you ask any of your family member(s) to accompany you during the clinical session(s)?
  ◦ Who did you ask, and why?

- What was the one thing you remember most, and why?
- What did you understand (from the clinician/genetic counsellor) about the implications of your results for your family members? (Probe: what considerations you have made, how the finding will affect your family members, and preventive action after confirming the finding)?
- Were there any difficulties or problems you encountered during the clinical session(s)?

1b. Is there anything else you want to say about this topic?

**Topic 2: Family**

2a. Who to tell

After you came back from the clinical session(s), how did you decide who you would tell about your diagnosis or test results, and why?

- Who did you decide not to tell, and why?
- Who do you consider your family?
- Other than family members at direct risk of [genetic condition], who else did you tell, and why?
- What were your principles/priorities in your decisions? (Probe: What are the different factors you took into consideration?)
- How did you tell them? (Probe: if there was a family chat group. Patterns of communication.)
- How did you decide what to do?
- Were you aware of family member(s)’ opinions about being told, i.e., if they would have wanted to know about their own risk?

2b. Telling family

Can you describe your experience(s) with family members* regarding disclosure of genetic risk information?

• Which family members have you informed (so far), and why?
  ◦ Were the reasons the same for everyone?
• What kind of information was given to those family members?
• How was information given to those family members? (e.g., in person, phone call, through another family member)
• For the family members who were told, what was their general reaction? (Probe: how families tell each other, positive and negative examples)
• Did you experience any issues/problems when telling family members?
  ◦ What was the nature of the problem?
  ◦ How did you decide what to do?
  ◦ What could be done to avoid such situations/problems in the future?

Is it acceptable if someone else tells your family members?

• Who would be acceptable? Who would not be acceptable? Can you tell me more?
• Do you have any feelings or concerns about this process?

Is it acceptable if HCP tells family members if a patient does not?

• Why? Why not?
• What are acceptable ways this can be done?

2c. Not telling family

Can you describe an experience when you did not tell a family member, either by choice or because you were prevented by circumstances?

• Did you experience any issues/problems?
  ◦ What was the nature of the problem?
  ◦ How did you decide what to do?
  ◦ What could be done to avoid such situations/problems in the future?

If the hereditary disease is not Lynch syndrome, but say high cholesterol, does your decision to inform family change?

2d. Family's right to know, or not

Do you think your family members should know about your genetic test results?

• Why, or why not? What factors do you consider important in your decisions?
• On the part of family members, do they hold any kind of right to know about your genetic test results?
• If they do have right(s), what is it based on?
• What is the extent of these right(s)?

Do you think it's important for you to know your family's medical history?

• Did you know much about your family's history? Were you inspired to find out more? Why?

2e. Views on genetic information

How do you see genetic information?

• What does it mean for you? Your family?
• Do you see genetic information as different from other types of medical information?
• Do you see it as personal? Or is it shared and ‘belonging’ to the family?
• Why do you see genetic information in this way?
• Do you have any thoughts, concerns regarding genetic information?
  o If genetic information is personal, does the family have any rights to the patient's genetic information? Under what circumstances?

• Does it matter who has this access?
• If genetic information is shared, what is access based on? Clinical need? Duty to warn/rescue? Familial obligation?
• How do you see genetic testing?
• Is your decision to take or decline based in any way on family?

Are you worried about the implications of having taken a genetic test?

2f. Is there anything else you want to add about telling genetic results to family members?
